# Tetranucleotide frequencies differentiate genomic boundaries and metabolic strategies across environmental microbiomes

**DOI:** 10.1128/msystems.01744-24

**Published:** 2025-07-08

**Authors:** Matthew Kellom, Maureen Berg, I-Min A. Chen, Ken Chu, Alicia Clum, Marcel Huntemann, Natalia N. Ivanova, Nikos C. Kyrpides, Supratim Mukherjee, T. B. K. Reddy, Simon Roux, Rekha Seshadri, Gitta Szabo, Neha J. Varghese, Tanja Woyke, Emiley A. Eloe-Fadrosh

**Affiliations:** 1 DOE Joint Genome Institute, Lawrence Berkeley National Laboratory 1666, Berkeley, California, USA; 2 Environmental Genomics and Systems Biology Division, Lawrence Berkeley National Laboratory 1666, Berkeley, California, USA; Agroscope Standort Reckenholz, Zurich, Switzerland

**Keywords:** bioinformatics, microbiome, environmental microbiology, microbial ecology, metagenomics

## Abstract

Microbiomes are constrained by physicochemical conditions, nutrient regimes, and community interactions across diverse environments, yet genomic signatures of this adaptation remain unclear. Metagenome sequencing is a powerful technique to analyze genomic content in the context of natural environments, establishing concepts of microbial ecological trends. Here, we developed a data discovery tool—a tetranucleotide-informed metagenome stability diagram—that is publicly available in the integrated microbial genomes and microbiomes (IMG/M) platform for metagenome ecosystem analyses. We analyzed the tetranucleotide frequencies from quality-filtered and unassembled sequence data of over 12,000 metagenomes to assess ecosystem-specific microbial community composition and function. We found that tetranucleotide frequencies can differentiate communities across various natural environments and that specific functional and metabolic trends can be observed in this structuring. Our tool places metagenomes sampled from diverse environments into clusters and along gradients of tetranucleotide frequency similarity, suggesting microbiome community compositions specific to gradient conditions. Within the resulting metagenome clusters, we identify protein-coding gene identifiers that are most differentiated between ecosystem classifications. We plan for annual updates to the metagenome stability diagram in IMG/M with new data, allowing for refinement of the ecosystem classifications delineated here. This framework has the potential to inform future studies on microbiome engineering, bioremediation, and the prediction of microbial community responses to environmental change.

**IMPORTANCE:**

Microbes adapt to diverse environments influenced by factors like temperature, acidity, and nutrient availability. We developed a new tool to analyze and visualize the genetic makeup of over 12,000 microbial communities, revealing patterns linked to specific functions and metabolic processes. This tool groups similar microbial communities and identifies characteristic genes within environments. By continually updating this tool, we aim to advance our understanding of microbial ecology, enabling applications like microbial engineering, bioremediation, and predicting responses to environmental change.

## INTRODUCTION

Throughout the course of Earth’s history, microbial populations have adapted to survive and grow in a wide range of environmental conditions. Every environment hosts a combination of physicochemical conditions, nutrient regimes, and community interactions that shape the fitness of specific taxa and their evolved metabolisms (1–5). The diversity of these adaptations is captured in the genomes of individual microbes that coalesce into environment-specific microbiomes, suited to their conditions (6). This means that environmental metagenomes collectively represent the genomic traits of microbial communities under a myriad of selective pressure gradients, and their genomes should reflect differences in those selective pressures (7, 8). In disturbance ecology, maintaining relatively constant alpha-diversity when microbial communities are exposed to selective pressures is referred to as microbiome stability (9, 10). It has been shown that similar environments harbor similar stable microbiomes (11–13) and stable microbiomes have been observed along environmental gradients (8, 14–17). However, the extent to which microbial populations and metabolic functions can be distributed across environmental conditions is often unclear, inhibiting our ability to predict population composition and metabolic activity from environmental context (8, 16, 18, 19).

Shotgun metagenomics offers a lens through which to capture the taxonomic and putative functional diversity of microbial communities, enabling analyses of microbiome ecological trends across different environment types at a global scale (13, 19–21). As metagenome databases continue to grow, meta-analyses can leverage data across thousands of disparate data sets (22–26). Despite the massive increase in sequence data across diverse environments, one key barrier for deriving cross-environment ecological insights has been the sparseness of contextual information or metadata (27–29). One source of metadata that is often required for data submission, and thus regularly associated with metagenome data sets, is environment ecosystem classification (30). While broad ecosystem classifications may orient researchers toward major taxonomy and metabolic processes expected within a sample (31), the interplay between microbial communities and their environments is multifaceted, leading to a level of complexity that can make direct comparisons and predictions challenging (32, 33).

Previous studies have utilized diversity metrics or functional profiling to differentiate microbiome composition across ecosystems (19, 34–36). Here, we hypothesized that tetranucleotide frequencies from unassembled metagenome data could be used as a proxy to evaluate ecosystem-specific microbiome composition and applied this analysis to over 12,000 environmental metagenomes. Tetranucleotides, composed of four DNA bases (*k*-mer size of *k* = 4), are universally shared components of metagenomes with usage patterns that have phylogeny-resolving specificity not seen in di- or trinucleotides (37–39). Their small size allows for a relatively low possibility of 256 combinations to compare between metagenomes, which can be further reduced to 136 when accounting for duplicate frequencies of reverse complements (40). Converting tetranucleotide counts into frequencies (percentages relative to all tetranucleotide counts summed) normalizes usage patterns to be independent of sequencing data size, with some potential biases still present due to variations in sequencing depth and coverage of lower abundance taxa (41). We frame tetranucleotide frequencies as components of metagenomes, analogous to elements as components of minerals. In the geosciences, mineral stability diagrams depict two conditions for a given set of minerals: (i) axes that show physicochemical conditions necessary for minerals to coexist at equilibrium and (ii) which minerals are stable and which are unstable along those axes (42). We group metagenomes with respect to sampling environment using the Genomes OnLine (GOLD) ecosystem classifications (30) and show that tetranucleotide frequencies can be used as a signature to map metagenome compositions in a tetranucleotide-informed metagenome stability diagram. The use of *k*-mer frequency counting has recently been shown to be effective for supervised learning analyses with respect to sampled ecosystems, and closely correspond to phylogenetically aware diversity metrics (43). We extracted ranked lists of gene annotations that displayed differential abundances across all ecosystem classifications, along with gene annotations that differentiated specific ecosystems. To facilitate future analyses, we have implemented the tetranucleotide-informed metagenome stability diagram as a service available on the Department of Energy (DOE), Joint Genome Institute’s (JGI), and Integrated Microbial Genomes and Microbiomes (IMG/M) platform (24) (img.jgi.doe.gov/cgi-bin/mer/main.cgi?section=AdvAnalytics&page=environmental), with direct access to only the plotting features at microbiomes.jgi.doe.gov.

## RESULTS

### Development of a tetranucleotide-informed metagenome stability diagram

**Fig 1:**
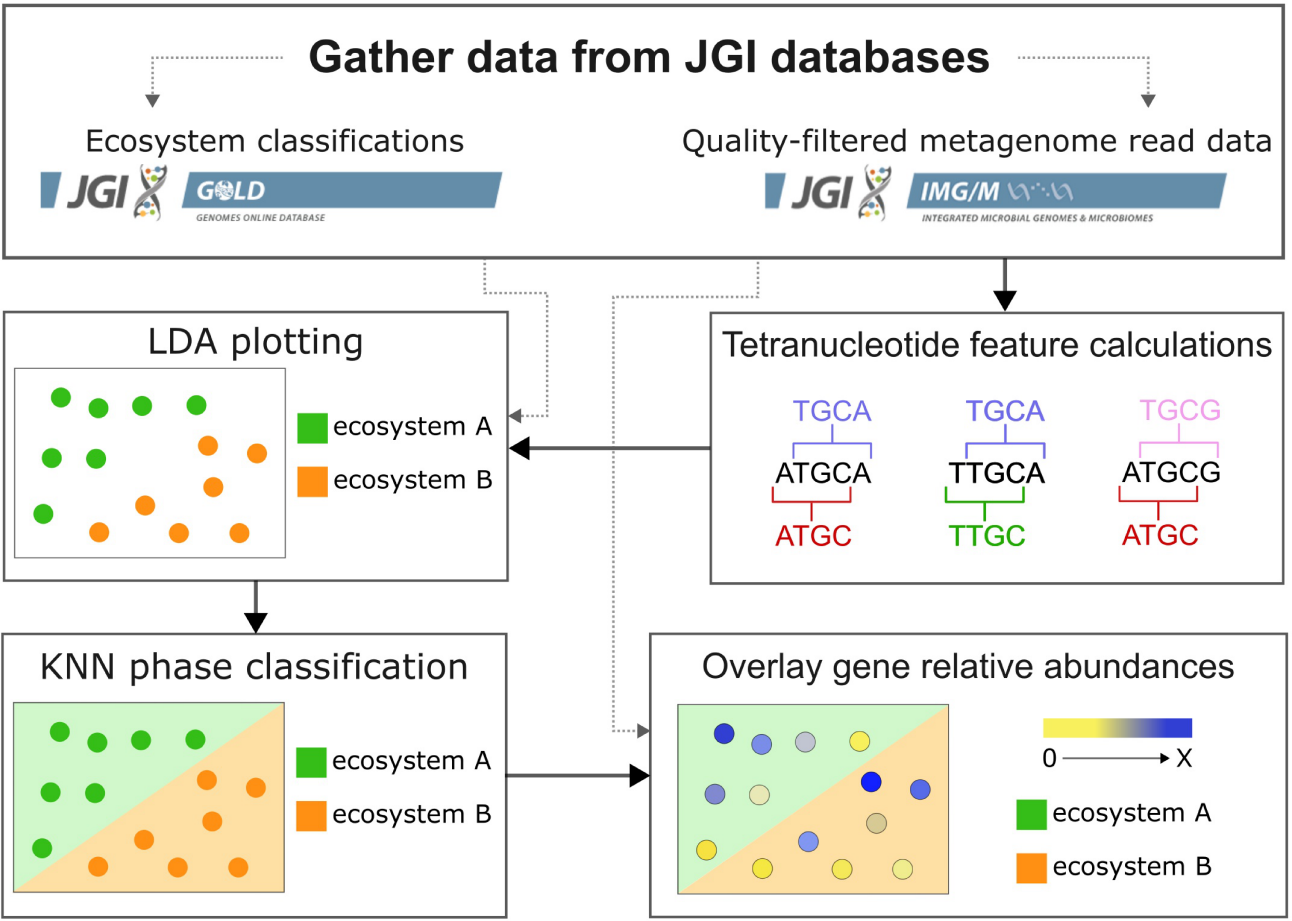
Overview of the analysis workflow to generate the metagenome stability diagram. First, quality-filtered and unassembled sequencing read data from IMG/M metagenomes were combined with ecosystem classifications from GOLD. Tetranucleotide frequencies were calculated for each metagenome (plotted as circles in this diagram), and then LDA plotting was applied with GOLD Ecosystem classification supervision. KNN was applied to LDA coordinates and GOLD Ecosystem classifications to map KNN Ecosystem phase classifications. Lastly, application of a heatmap overlay for IMG/M gene annotation relative abundances was applied.

Microbiomes from diverse ecosystems can be differentiated based on diversity metrics and unique functional profiles (19, 34–36). We leveraged tetranucleotide frequencies combined with linear discriminant analysis (LDA) dimensionality reduction and k-nearest neighbor (KNN) classification to generate a tetranucleotide-informed metagenome stability diagram for 12,063 metagenomes (Fig. 1). These metagenomes span diverse natural environments, with the majority sampled from soil (5,236), marine (2,761), and freshwater (2,481) ecosystems and have been manually curated with the five-level GOLD Ecosystem classification scheme (30) (File S1). We assumed that all naturally occurring metagenomes will contain all possible tetranucleotide combinations as universal basic shared components (38). Both tetranucleotide frequencies and ratios of tetranucleotide frequencies were used for metagenome analysis (File S4), as described in Methods.

**Fig 2:**
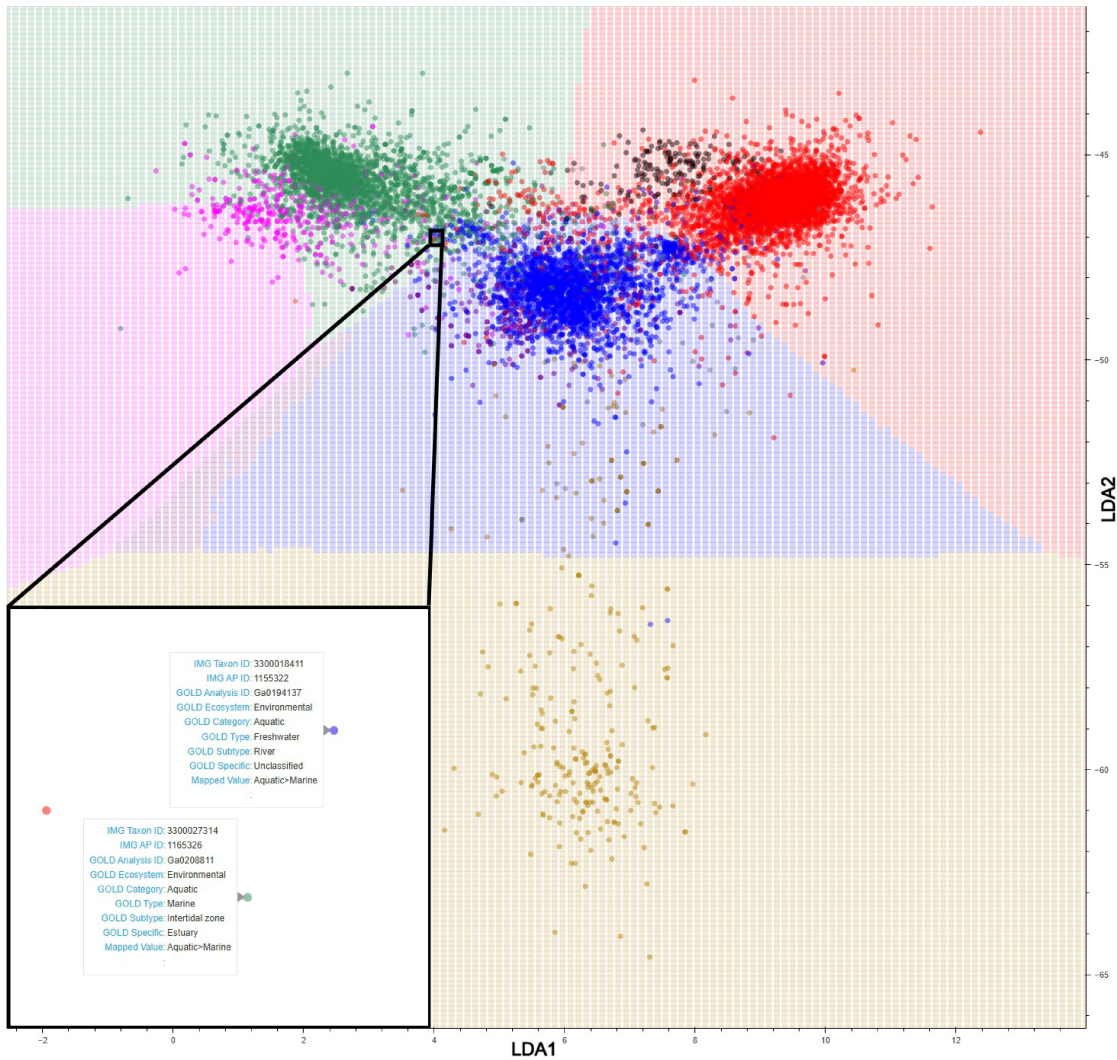
Metagenome stability diagram created with LDA dimensionality reduction of tetranucleotide frequencies and ratios of tetranucleotide frequency and KNN application. Each data point represents a metagenome, colored according to their GOLD Ecosystem Type classification, with the most abundant being Soil (red), Freshwater (blue), Marine (green), Non-marine saline and alkaline (pink), Thermal springs (gold), Plant litter (black), and Deep subsurface (purple). A full list of metagenomes used is included in File S1. A zoomed-in panel (bottom left) with IMG and GOLD details is used to demonstrate each point in the diagram as an individual metagenome. Background regions represent KNN Ecosystem classification stability phases of the same color scheme as the metagenome data points, with the exception of Plant litter, which does not have a mapped phase due to lack of KNN support. The counts of plotted metagenomes from each KNN Ecosystem classification are available in File S1. Metagenomes that have a matching GOLD ecosystem type data point color and KNN ecosystem classification phase background color have tetranucleotide compositions that resemble other metagenomes of the same GOLD Ecosystem Type, as derived by LDA with KNN classification. Metagenome data points that plot over differing KNN Ecosystem classification phases have tetranucleotide compositions that more closely resemble the differing phase rather than their own GOLD Ecosystem Type classification.

Since the number of tetranucleotide features for each metagenome creates far more dimensions than can be visualized (9,316 tetranucleotide frequencies and ratios of frequencies), dimensionality reduction is required for plotting in a two-dimensional space. We used LDA dimensionality reduction to supervise *x*- and *y*-coordinate plotting of metagenomes according to both their tetranucleotide composition and GOLD Ecosystem classification labels. LDA seeks to project multidimensional data (tetranucleotide features) into axes that maximize the distance between labeled groups (GOLD Ecosystem classifications) and minimize variation within groups of the same label (44). Once metagenomes were plotted with LDA *x*- and *y*-coordinates, we used the KNN algorithm to delineate ecosystem classification regions within the two-dimensional space (further described in Methods), which could then be used to evaluate metagenomes sampled along environmental gradients and determine which metagenomes and metagenome properties approach ecosystem classification boundaries (Fig. 2). The resulting interactive metagenome stability diagram is hosted on the IMG/M site as a publicly available resource (img.jgi.doe.gov/cgi-bin/mer/main.cgi?section=AdvAnalytics&page=environmental), with direct access to only the plotting features at microbiomes.jgi.doe.gov.

Each data point of the metagenome stability diagram in Fig. 2 represents an individual metagenome, and the background shows KNN Ecosystem classification boundaries, with colors representing the metagenomic space of GOLD Ecosystem Type classifications: “Terrestrial>Soil” (red), “Aquatic>Freshwater” (blue), “Aquatic>Marine” (green), “Aquatic>Non-marine Saline and Alkaline” (pink), “Aquatic>Thermal springs” (gold), and “Aquatic>Deep Subsurface” (purple). Metagenome counts of these classifications as well as finer scale GOLD Ecosystem counts are available in File S1. The KNN Ecosystem classification boundaries between each of these metagenomic phases (e.g., gold to blue meaning “Aquatic>Thermal springs” to “Aquatic>Freshwater”) represent KNN Ecosystem Type transitions, where the tetranucleotide content shifts from a composition resembling metagenomes with one GOLD Ecosystem Type classification to metagenomes of another GOLD Ecosystem Type. The large condensed clusters of data points represent metagenome compositions that are commonly found for the particular KNN Ecosystem classification phase that they are plotted in, which often represent more commonly sampled environments. These metagenomes remain largely unseparated by LDA projection of within-group variation and form a “central” grouping. Metagenome data points that are “peripheral” to the central core metagenomes represent uncommon tetranucleotide compositions and often originate from lesser sampled GOLD Ecosystem Subtype environments.

### A view into community transitions across ecosystems

Insight into environmental microbiome properties that help to define both central and peripheral metagenome classifications could help reveal microbe-environment interactions that shape our biosphere (45). The transition boundaries between KNN Ecosystem classification phases represent KNN-classification ambiguity, where metagenomes plotted near these boundaries can be labeled with either classification. Presumably, this ambiguity reflects environmental conditions approaching high similarity, but we cannot be certain without detailed environmental metadata which is currently sparse for our collected metagenomes. Since the exact conditions or microbial compositions for which an environment classification begins to transition to another is elusive, we cannot define which metagenomes are within the fringe space between KNN Ecosystem classification phases with clear delineations. However, exploring these transition-associated GOLD Ecosystem classifications more specifically, metagenomes plotted near classification transition boundaries are often sampled from environments along ecosystem gradients, such as “Wetlands,” “Estuaries,” and “Sediment,” where two environments mix (e.g., “Wetlands” as a mix between “Soil” and “Freshwater”). The boundary between “Marine” and “Non-marine Saline and Alkaline” classifications, in particular, appears the least resolved from our collection of metagenomes. Intuitively, these metagenomes near the “Aquatic>Marine” and “Aquatic>Non-marine Saline and Alkaline” boundary would likely be sampled from marine coastal mixing environments, and in fact, many are sampled from “Coastal,” “Inlet,” and “Intertidal” GOLD Ecosystem Types where estuarine mixing occurs (46). In Fig. 2, the KNN Ecosystem classification of an “Aquatic>Deep subsurface” phase did not contain any plotted metagenomes, but presence of a KNN classification phase implies most nearby metagenomes are annotated as such. KNN classification phase plotting is further described in Methods as k-nearest metagenome GOLD Ecosystem Type classification color assignment to the background phase space. Indeed, a large number of “Aquatic>Deep subsurface” annotated metagenomes within the “Aquatic>Freshwater” phase are skewed toward the lower left corner, in the direction of the “Aquatic>Deep subsurface” phase. The “Aquatic>Deep subsurface” phase borders the “Aquatic>Marine,” “Aquatic>Non-marine Saline and Alkaline,” “Aquatic>Freshwater,” and “Aquatic>Thermal springs” phases, which suggests that any hypothetical metagenomes in the “Aquatic>Deep subsurface” phase may resemble a mixing of microbial communities from those environments.

### Ranked list of protein-coding genes that differentiate across ecosystems

**Fig 3:**
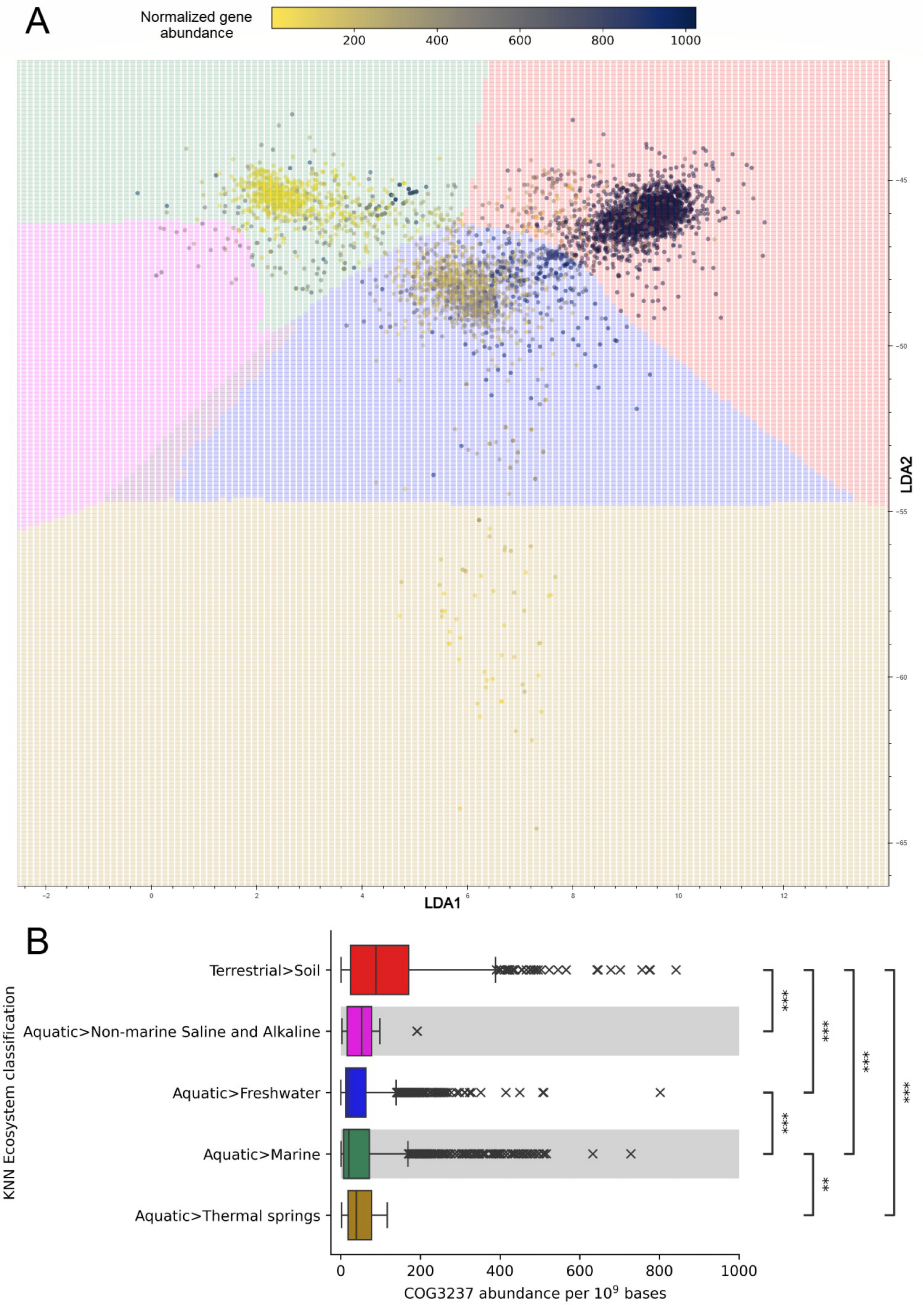
Ecosystem classification distribution of COG3237 normalized abundance. (**A**) Metagenome stability diagram showing gene abundances of COG3237, uncharacterized conserved protein YjbJ, normalized per 10^9^ assembled bases. The Matplotlib (47) reverse cividis colormap was applied to plotted abundances, with a perceptually uniform sequential scale from low abundance (yellow) to high abundance (dark blue). Metagenomes that are missing COG3237 annotation are hidden and not assigned a color. Background color assignment and plotting coordinates are the same as in Fig. 1. (**B**) A boxplot summary of COG3237 normalized abundances shown in (**A**). Welch’s ANOVA Games-Howell post hoc test was used to calculate pairwise statistical significance between non-equal variance KNN ecosystem classification groups; *P ≤ 0.05; **P ≤ 0.01; ***P ≤ 0.001.

Metagenomes within each KNN Ecosystem classification phase represent different microbial community compositions with differing metabolic capabilities. In addition to GOLD Ecosystem classification annotations, metagenomes have been processed through the IMG annotation pipeline, providing functional information *via* protein-coding gene identifiers. Gene relative abundances, normalized to the amount of assembled bases, are calculated for metagenomes and can be grouped according to KNN Ecosystem classifications of Fig. 2 and/or GOLD Ecosystem classifications. In the IMG/M interactive tool, gene abundances were overlaid on metagenome data points with a heatmap-like color scheme and show patterns of metabolic capabilities in the context of their KNN Ecosystem classification phase. The heatmap color range can be manually adjusted with the “Heat” slider. As an example, Fig. 3A shows normalized gene abundance heatmap values for COG3237, an uncharacterized conserved protein subunit (YjbJ). When summarized as a box plot (Fig. 3B), COG3237 has significantly greater abundances in the “Terrestrial>Soil” KNN Ecosystem classification, suggesting an ecosystem-specific role for this uncharacterized protein.

Using a random forest model trained on the metagenome GOLD Ecosystem Type classifications as well as our KNN Ecosystem Type classifications, with protein-coding annotations, we extracted ordered lists of Clusters of Orthologous Genes (COGs) (48), Enzyme Commission (EC) (49), Kyoto Encyclopedia of Genes and Genomes Orthology (KO) (50), and European Bioinformatics Institute protein families (Pfam) (51) gene identifiers that exhibit differential abundance across all ecosystems and identifiers that differentiated specific ecosystems classifications (File S2). The gene identifiers that best differentiate each GOLD Ecosystem Type classification and plotted KNN Ecosystem Type classification, either because they are abundant or sparse, are presented in Table 1. These ranked gene identifiers help to confirm the separation of metagenomes based on genomic properties evolved from life-environment interactions and metabolic properties that confer selective advantage or disadvantage.

**TABLE 1:** Top gene identifiers that are differentiated by each ecosystem type from GOLD classifications or ecosystem type classification from our KNN analysis and their gene annotation description^*a*^

Ecosystem type	Most differential gene identifier	Annotation description
GOLD: Terrestrial>Soil	COG3237 abundance	Uncharacterized conserved protein YjbJ, UPF0337 family
GOLD: Aquatic>Thermal springs	COG1483 abundance	Predicted ATPase, AAA+ superfamily
GOLD: Aquatic>Non-marine Saline and Alkaline	COG1191 sparsity	DNA-directed RNA polymerase specialized sigma subunit FliA
GOLD: Aquatic>Marine	COG4208 sparsity	ABC-type sulfate transport system, permease component CysW
GOLD: Aquatic>Freshwater	COG3329 abundance	Na+-dependent bicarbonate transporter SbtA
KNN: Terrestrial>Soil	COG3237 abundance	Uncharacterized conserved protein YjbJ, UPF0337 family
KNN: Aquatic>Thermal springs	KO:K09127 abundance	CRISPR-associated protein Cmr3
KNN: Aquatic>Non-marine Saline and Alkaline	PF15613 abundance	WSD, D-TOX E motif
KNN: Aquatic>Marine	KO:K05801 abundance	DnaJ like chaperone protein
KNN: Aquatic>Freshwater	KO:K09797 abundance	uncharacterized protein

^*a*^ A full list of annotated genes is available in File S2.

The prevalence of COG3329 and sparsity of COG4208 were the top genes differentiating “Aquatic>Freshwater” and “Aquatic>Marine” GOLD classifications, respectively. The Na^+^-dependent bicarbonate transporter SbtA (COG3329) and the permease component of an ABC-type sulfate transport system CysW (COG4208) are both nutrient transporters that indicate metabolism specificity in response to the environment. The high abundance of *sbtA* in freshwater environments suggests a demand for bicarbonate uptake, which is consistent with the observation of the ubiquitous presence of this gene in cyanobacteria genomes often found in freshwater ecosystems (52). The cause of low abundance of *cysW* in marine environments is more difficult to hypothesize, since low abundances in genomes could be due to alternative transporter presence or a lower need for sulfate uptake transport systems in marine environments (53). When considering KNN Ecosystem Type classification of metagenomes, “Aquatic>Freshwater” and “Aquatic>Marine” were most differentiated by the prevalence of genes for the uncharacterized protein KO:K09797 and the DnaJ like chaperone protein KO:K05801, respectively. KO:K05801 (gene *djlA*) is a homolog of protein DnaJ and is involved in the Rcs pathway, a signaling cascade that responds to outer membrane and peptidoglycan stress induced by osmotic shock (54). Most of the metagenomes with high counts of KO:K05801 are from marine coastal mixing environments, where there are likely microbial populations adapted to osmotic stress conditions. The uncharacterized protein KO:K09797 is relatively understudied but is linked to hypothetical protein COG2859, which was the 13th most differential gene identifier for the “Aquatic>Freshwater” KNN Ecosystem Type classification. One study identified COG2859 in freshwater Actinobacteria lineage acI, one of the most abundant microorganisms in freshwater lakes, but no other lineages were found to contain this annotation in their work (55).

As previously shown in Fig. 3, *yjbJ* is highly abundant in soil environments with a function that is largely unknown, but its expression is increased under osmotic stress (56). *yjbJ* was also one of the top gene identifiers to show differential abundance across all ecosystem classifications, with high abundances in soil ecosystems and a gradual decrease across freshwater to low abundances in marine ecosystems (Fig. 3), seemingly in agreement with reports of *yjbJ* having a potential role in desiccation resistance (57). The predicted ATPase of the AAA+ superfamily (most differential in thermal spring GOLD classification environments) and *fliA* (most differential in non-marine saline environments) are less specific toward metabolic processes and instead may be indicative of highly abundant taxa and/or taxa that contain many copies of these genes. Similarly, clustered regularly interspaced short palindromic repeat (CRISPR)-associated protein Cmr3 and Williams-Beuren syndrome DDT (WSD), D-TOX E motif, that are the most differential gene identifiers in “Aquatic>Thermal springs” and “Aquatic>Non-marine Saline and Alkaline” KNN classifications may also signify abundant taxa. Cmr3 participates in taxa-specific anti-phage defense (58), and WSD is a widely conserved protein found in plants (59), which is not unexpected since the metagenomes with the greatest abundances in “Aquatic>Non-marine Saline and Alkaline” are labeled as microbial communities from algal ponds.

### Investigating metabolic interactions with the environment

**Fig 4:**
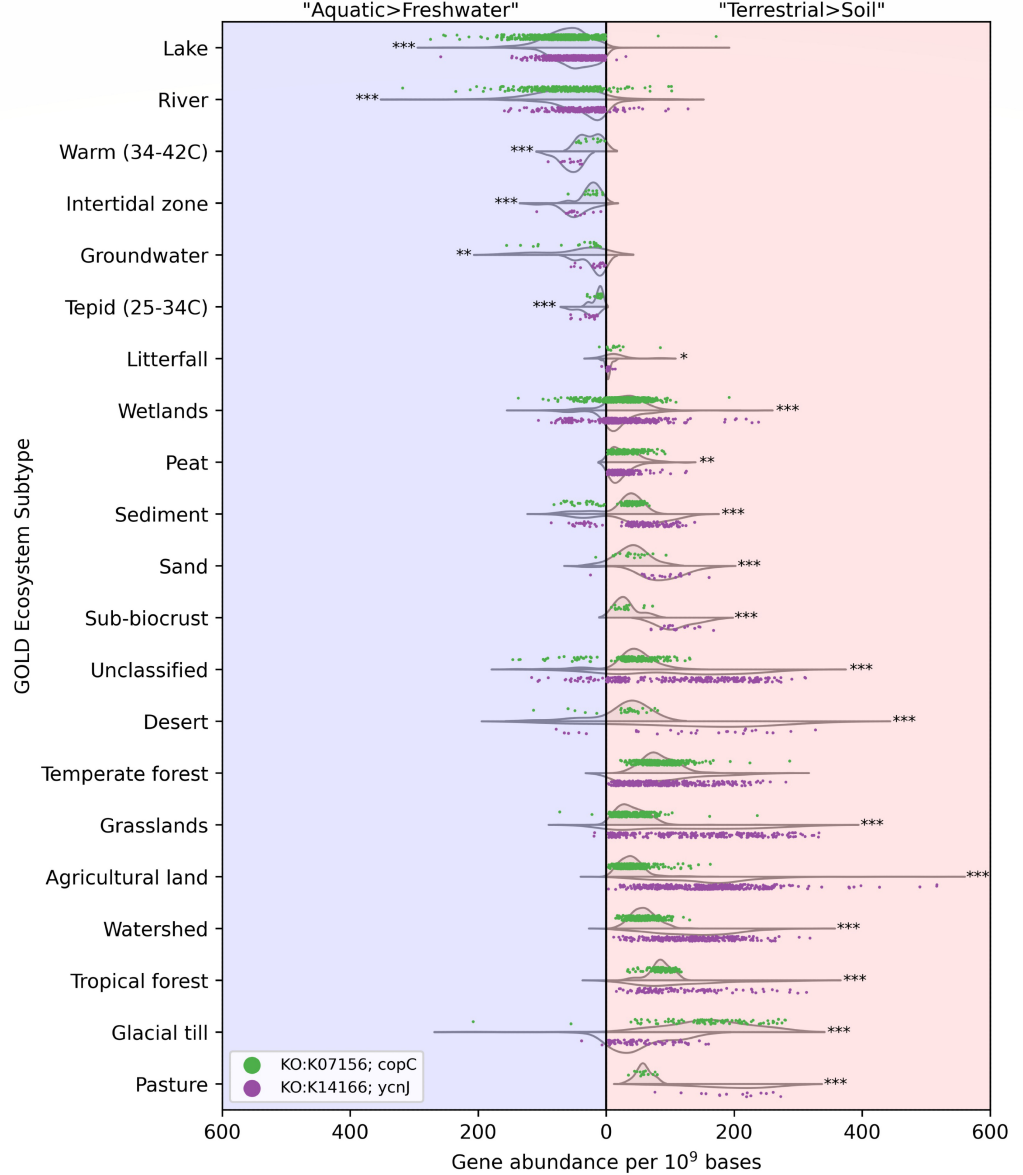
Comparison of KO:K07156 (*copC*) (green points) and KO:K14166 (*ycnJ*) (purple points) metagenome gene abundances relative to 10^9^ assembled bases across GOLD ecosystem subtypes with a diverging *x*-axis. Metagenomes within the “Aquatic>Freshwater” (blue background, left of vertical line at *x* = 0) and “Terrestrial>Soil” (red background, right of vertical line at *x* = 0) KNN Ecosystem classification phases of Fig. 2 have their *copC* and *ycnJ* gene abundance data points plotted within corresponding *x*-axis range here. Metagenomes with no abundance data available for either gene are not included in the plot. Only GOLD ecosystem subtypes with >10 representative metagenomes are included, totaling 3,865 metagenomes across 21 subtypes. To better examine data point distribution in crowded *x*-axis ranges, split violin plots highlight data point density for KO:K07156, (*copC*) (top-side violin) and KO:K14166, (*ycnJ*) (bottom-side violin) gene abundances within each GOLD Ecosystem Subtype. Two-tailed *t*-test statistical significance of each KO:K07156 (*copC*) and KO:K14166 (*ycnJ*) pairing was calculated for “Aquatic>Freshwater” and “Terrestrial>Soil” data points separately; *P ≤ 0.05; **P ≤ 0.01; ***P ≤ 0.001.

The use of protein annotations in a metagenome stability diagram enables visualization of successful metabolic strategies. Using protein-coding annotations to estimate environmental nutrient concentrations and speculate on nutrient/energy capture is established in microbial ecology (2, 3, 60, 61). For certain substrates, cells must maintain the proper balance of flux to meet metabolic demands while also avoiding toxicity (62). Copper is a prime example, because as a metal cofactor, it is required for many enzymes to function while also being toxic if internal concentrations become too great (63). The distribution of *ycnJ* (KO:K14166), a copper importer protein subunit (64), and *copC* (KO:K07156), a copper-resistance protein subunit (64–66), across “Aquatic>Freshwater” and “Terrestrial>Soil” KNN Ecosystem classification phases highlights differing metabolic strategies for copper flux balance within each environment (Fig. 4). These two genes encode for proteins contained within the same COG identifier (COG2372) and highlight the complicated and understudied mechanisms of copper homeostasis. YcnJ is a homolog of the CopCD dimer and functions as a copper importer in genus *Bacillus* (64, 67), and CopC has an unclear mechanism for imparting copper resistance with potential roles in copper efflux, sequestration, and import (66, 68). While these two genes do not provide a complete view of copper homeostasis (69), Fig. 4 shows the prevalence of potential copper resistance *via* CopC relative to YcnJ-mediated copper influx for specific GOLD Ecosystem Subtypes split across two KNN Ecosystem classification phases. In the “Aquatic>Freshwater” phase, higher abundances of *copC* are found in Lake, River, and Groundwater subtypes, suggesting microbial populations that are susceptible to copper toxicity and possibly exist in copper-replete conditions, where copper resistance is beneficial. GOLD Ecosystem Subtypes of Warm (34-46C), Tepid (25-34C), and Intertidal zone had higher abundances of *ycnJ*, indicating a demand for copper among those populations, which likely includes the heat and salt-tolerant genus *Bacillus* commonly found in soil and freshwater environments (70, 71). Despite relatively high counts of metagenomes within the “Aquatic>Freshwater” phase, Wetlands, Sediment, and Unclassified GOLD Ecosystem Subtypes had no difference between *copC* and *ycnJ* abundances. In the “Terrestrial>Soil” phase, all GOLD Ecosystem Subtypes with a greater number of metagenome samples than in the “Aquatic>Freshwater” phase had higher abundances of *ycnJ*, except Peat, Glacial till, and Temperate forest. Higher abundances of *ycnJ* suggest that soil populations are generally in need of copper supply. Or relative to soil, freshwater microbiomes contain greater abundances of taxa with alternative copper uptake mechanisms. Conversely, Glacial till and Peat had higher abundances of *copC* and, along with the Lake, River, and Groundwater subtypes of the “Aquatic>Freshwater” phase, may contain populations that benefit from copper resistance. Removing or adding copper in these ecosystems would likely create instability for these microbiomes, triggering a metagenome compositional shift (72) toward a nearby region of their KNN Ecosystem classification phase that has tolerable copper conditions.

**Fig 5:**
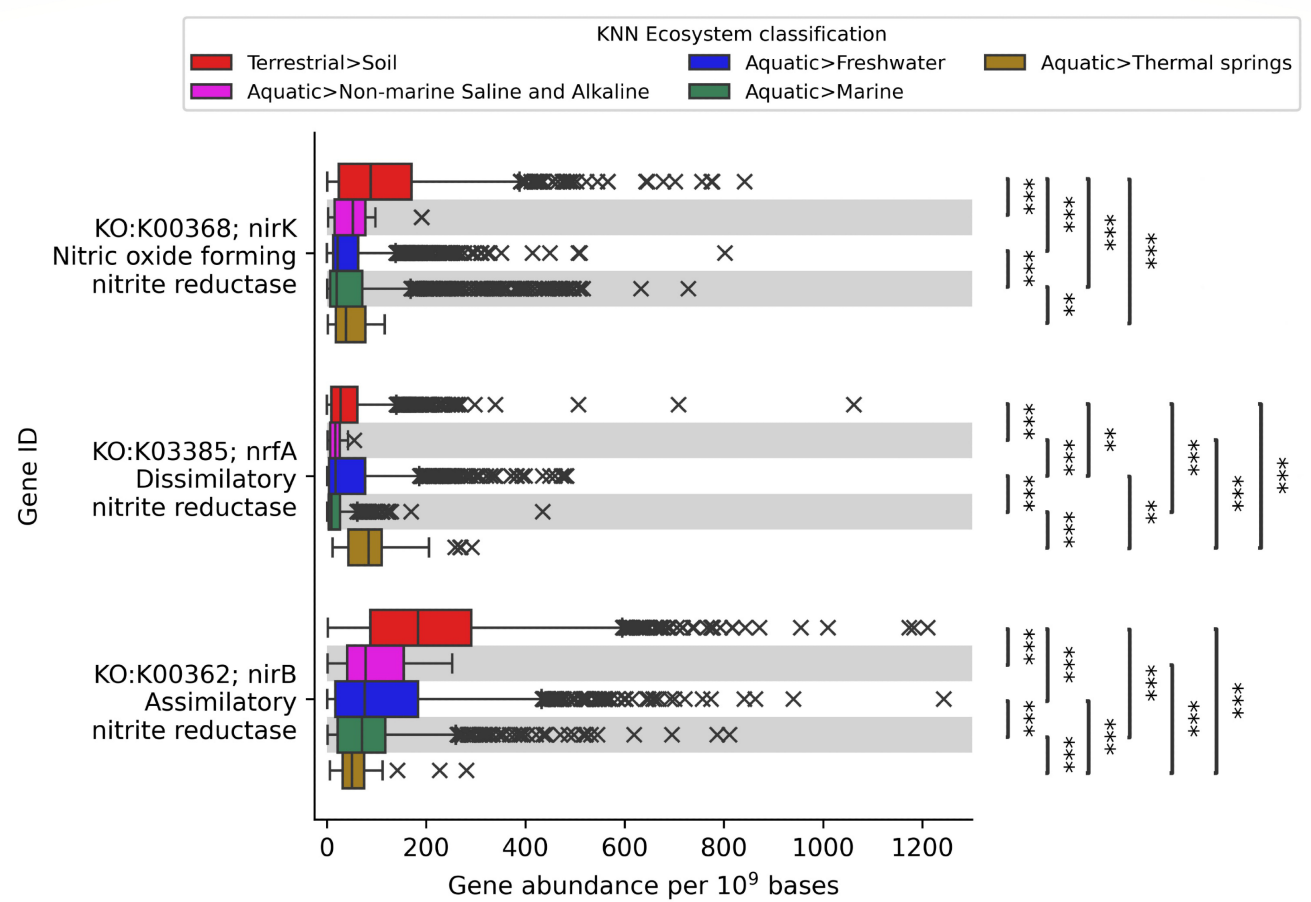
Nitrite reductase box plot showing normalized gene abundances for KO:K00368 (nirK), KO:K03385 (nrfA), and KO:K00362 (nirB) across KNN Ecosystem classification metagenomes of the metagenome stability diagram of Fig. 2. Welch’s ANOVA Games-Howell post hoc test was used to calculate pairwise statistical significance between non-equal variance KNN Ecosystem classification groups; *P ≤ 0.05; **P ≤ 0.01; ***P ≤ 0.001.

We also used protein-coding annotations of metagenomes to decipher patterns of nutrient and elemental cycling across ecosystem classifications. The nitrogen cycle, which includes both oxidative and reductive pathways along with assimilatory and dissimilatory nitrogen usage (73), is extensively studied based on metagenomic data (74–76). Nitrate reduction to nitrite is the first step of multiple reducing pathways with differing end products, each with their own microbiome implications. Figure 5 explores the metagenomic patterns of nitrite reduction, looking at the fate of nitrite in the global nitrogen cycle. *nirK* (KO:K00368) is a gene marker for copper-containing nitric oxide forming nitrite reductase, involved in a process called denitrification in anaerobic environments, reducing nitrite to gaseous nitrous oxide (77). Denitrification will eventually produce N_2_O and N_2_ gas, which is no longer a fixed-nitrogen source and lost to the atmosphere. *nirK* abundances were highest in soil metagenomes with sparse abundance peaks in aquatic environments having high sediment contact, such as rivers, floodplains, intertidal zones, and hydrothermal vents, which may represent anaerobic conditions (File S3). *nrfA* (KO:K03385) is a gene marker for heme-containing dissimilatory nitrite reductase that reduces nitrite to ammonium, using nitrite as an electron acceptor and keeping nitrogen fixed in the environment (78). In addition to nitrogen fixation and organic nitrogen catabolism, dissimilatory nitrite reduction supplies bioavailable ammonium to the environment, which feeds nitrogen metabolism in oxidative conditions. *nrfA* abundances were generally highest in thermal spring environments with abundance peaks in restoration grassland soil environments with agricultural history, although the highest abundance is from an Antarctica dry valley desert soil metagenome. In the case of restoration grassland soil metagenomes, the high abundance of *nrfA* is likely due to the regrowth of native perennial leguminous plants after fertilizer soil amendments have ceased (79), which selects for symbiotic nitrogen-fixing bacteria. Assimilatory nitrate reductase marker *nirB* (KO:K00362) also converts nitrite to ammonium, which is then in turn used for the synthesis of biomolecules (78). *nirB* abundances were also highest in soil environments but more prevalent across aquatic environments than *nirK* and *nrfA*. Grouping metagenomes by genomic content and highlighting metabolic capabilities helps to decipher the fate of global nutrient cycling across ecosystem classifications. In the case of nitrite, in Fig. 5, we have observed metabolic abundance patterns that drive the bioavailability of nitrogen as it cycles globally.

## DISCUSSION

This work aims to determine genome-derived boundaries that separate metagenome ecosystem classifications and help reveal microbe-environment interactions that shape our biosphere. We use tetranucleotide compositions and ecosystem classification observations to delineate boundaries between metagenome groups, similar to the use of chemical compositions delineating mineral samples in mineral stability diagrams (42). Tetranucleotides are universally shared components of (meta)genomes (37), which means that any distance between metagenomes within our metagenome stability diagram is a result of varying tetranucleotide frequencies. Metagenome samples can be thought of as aggregates of microbial populations, and since tetranucleotide frequencies are taxonomy-specific (38, 39), microbial population composition is the determining factor for tetranucleotide frequency composition and metagenome coordinates within Fig. 2. In contrast, other *k*-mer-based implementations of determining metagenomic distance can be supported by comparing their fraction of shared *k*-mers (80, 81). Although both methods of determining metagenomic distance are based on microbial community composition, the distance between hypothetical metagenomes with no shared *k*-mers is incalculable, which is increasingly possible with increasing *k*-mer size and taxonomic distance (82). While *k*-mer sizes larger than tetranucleotides may increase taxonomic specificity for identification and diversity metrics (43, 82), microbial community taxonomy assignment is not needed for our purposes of metagenome plotting.

One assumption of our analysis is that each metagenome represents a stable microbiome in its plotted KNN Ecosystem classification phase, i.e., having a microbial composition that is adapted to its environmental conditions. Within each classification phase, however, multiple variations of microbiome stability can exist, since each microbial community has a past history of adaptation to niche perturbations (32, 83–87). This environmental context contributes to the clustering spread that can be observed in all classification phases and may be influenced by unusually sampled microbial populations. Likewise, convergent evolution processes may lead to functionally similar microbial compositions with distinct tetranucleotide frequencies, decreasing cluster cohesion. Interestingly, the “Aquatic>Thermal springs” classification phase is relatively less clustered than all others, which could be due to the heterogeneous environments classified as thermal springs and the range of potential metabolisms supported by varying mineral and nutrient availability (15, 88). Metagenomes are also less clustered near KNN Ecosystem classification phase transitions, which may be representative of varying environmental conditions along a gradient between GOLD Ecosystem Types. While phase transitions represent environment mixing, the physicochemical properties are constant enough to drive the formation of stable microbial compositions (6, 32). Decreased clustering near phase transitions could also be a result of lacking metagenome data sampled from mixing environments. Although the “Aquatic>Deep subsurface” phase currently does not contain metagenome data points, as more metagenomes are plotted into the metagenome stability diagram, the coordinates of this phase may migrate to a region honing in on tetranucleotide composition distinction from “Aquatic>Freshwater,” reflecting environmental physicochemical properties. Similarly, “Terrestrial>Plant litter” annotated metagenomes appear to be nearing tetranucleotide composition distinction from “Terrestrial>Soil” metagenomes, but have not yet reached a density within the Fig. 2 LDA coordinates such that KNN classification of a “Terrestrial>Plant litter” phase has been plotted. Areas of the metagenome stability diagram that do not yet contain plotted metagenomes represent hypothetical microbial community compositions from niche environments that have yet to be sampled, or conditions that are beyond the limits for life (89, 90). Considering metagenomes that are nearby on the metagenome stability diagram, areas that are so far unrepresented by data points may guide future sampling expeditions.

Recently, the clustering of KO gene identifiers from IMG/M metagenomes identified metagenome clusters reflecting terrestrial, aquatic, and anaerobic ecosystems (19). As part of their analysis, three clusters were differentiated by functional markers and guanine-cytosine (GC) content. Remarkably, their study, which clustered metagenomes by protein-coding annotations and resulted in sequence content differentiation, identified similar top ecosystem-specific differentiating gene markers in an approach that is the opposite of ours using clustering with unassembled sequence content (tetranucleotides from quality-filtered sequence reads) (File S2). Across all ecosystems, Flinkstrom et al. found two non-homologous end-joining DNA repair genes positively correlated with their finding of GC content differentiation among clustered metagenomes, *ligD* and *ku*. From our analysis using GOLD Ecosystem Type annotations, the *ku* gene identifier COG1273 was the fourth highest overall differentiating gene identifier, with KO:K10979 (KEGG gene identifier for *ku* as in Flinkstrom et al. [19]) as the 514th highest, out of 45,536 total gene identifiers. *ligD*, as KO:K01971, was the 336th highest gene identifier overall in our analysis, and 283rd as COG1793. The functionality of genes in these rankings toward microbial population adaptation to specific environments is largely unvalidated. The ranking of genes as differentiating across and within ecosystem classification could be an important guiding tool for future studies characterizing proteins of unknown function or refining concepts of protein function and metabolism dispersal.

### Conclusion

Metagenome ecosystem stability implies that microbial populations are able to acquire, metabolize, and excrete chemical compounds required for growth, to which their lineage has become adapted (91). Understanding these processes relies on comparison of both biological and environmental components (15, 17). Any environmental conditions that limit metabolic processes help to create ecosystem classification boundaries within our metagenome stability diagram, since microbial composition will be forced to adapt (92, 93). There is also an expectation that if nutrients exist with favorable environmental conditions, a microbial population will grow and consume those nutrients (94, 95). With this in mind, the metabolic potential of metagenomes in the form of protein-coding annotations provides insight on environmental conditions and nutrient flux. Future metagenomes sequenced, assembled, and annotated by the Joint Genome Institute will be analyzed with the methods laid out here and contribute to future annual updates to the interactive metagenome stability diagram tool in IMG/M. This work could help lead to a future of microbiome science where microbial population compositions can be predicted based on initial and/or perturbed environmental conditions and possibly have implications for microbiome engineering.

## MATERIALS AND METHODS

Publicly available metagenomes sequenced at the JGI and added to the IMG database before 10 April 2024 (date of collection) were considered for this analysis (24). This data collection criteria yielded 15,208 metagenome data sets labeled as “Metagenome Analysis” as their GOLD Analysis Project Type (96). Metagenomes that had a GOLD Ecosystem classification of “Engineered” or “Host-associated” or had a GOLD Ecosystem Type classification of “Nest” were removed from our metagenome collection for non-natural or host-restricted environment properties that would alter the interpretation of tetranucleotide frequencies plots as reflecting physicochemical pressures. After data filtering, the total number of included metagenomes was 12,063, with GOLD Ecosystem Categories and Types that can be found in File S1.

From the included metagenome data sets, filtered sequencing reads following the standard operating procedure of the DOE-JGI Metagenome Annotation Pipeline (97) were analyzed for tetranucleotide counts with KMC (version 3.1.1) (98). For each metagenome, tetranucleotide counts were converted into frequencies (e.g., each 4-mer count divided by the sum of all 4-mer counts of that metagenome) and ratios of those tetranucleotide frequencies (e.g., each 4-mer frequencies divided by each of the other 4-mer frequencies) (File S4). Tetranucleotide frequencies and ratios of frequencies were used as features for LDA dimensionality reduction using Scikit-learn (v1.5.2) (99) to plot metagenomes in a two-dimensional space with reference to their GOLD Ecosystem Type classification. Including the ratios of tetranucleotide frequencies as features for our metagenome LDA dimensionality reduction allows additional vector coefficients (100) to be utilized for roughly estimating relative abundances between taxa/metabolisms. LDA is a supervised algorithm that calculates *x*- and *y*-coordinates that maximize group separation and minimize intra-class variation (100). Metagenome groups were defined as “GOLD Ecosystem Category>GOLD Ecosystem Type” (File S1). An LDA training/testing data split of 80%/20% was used to assess accuracy, precision, and *F*1-score statistics, which was 0.91 for each. This LDA model was then applied to all of our selected metagenomes. Once metagenome *x*- and *y*-coordinates were plotted, KNNs were used to define metagenome stability phases as KNN Ecosystems classifications. A grid of square data points plotted in the background of Fig. 2 and 3A, as well as the online tool, was assigned a KNN Ecosystem Type classification based on the GOLD Ecosystem Type classification of k-nearest metagenomes and is used to visualize KNN Ecosystem classification boundaries. A value of *k* = 500 nearest metagenomes was chosen based on diminishing accuracy, precision, and *F*1-score statistics when increasing *k*, using a training/testing data split of 80%/20% (Fig. S1). Values of *k* < 500 showed evidence of overfitting with GOLD Ecosystem Type classification boundaries deviating to include outlier data points. Overfitting at lower *k* values is not surprising since KNN as a classifier is prone to overfitting due to noise sensitivity at lower values of *k* and can be ameliorated by employing larger *k* values (101). The metagenome stability phases that are plotted with KNN in Fig. 2 are “Terrestrial>Soil,” “Aquatic>Freshwater,” “Aquatic>Marine,” “Aquatic>Non-marine Saline and Alkaline,” “Aquatic>Thermal springs,” and “Aquatic>Deep Subsurface.” Data visualization was inspired by Kaushik (102), showing how to visualize classifier decision boundaries. Visualization was done with the Python-based customizable plotting tool Bokeh (v3.4.3) (103). The code for the construction of the interactive tool at microbiomes.jgi.doe.gov is available at github.com/mkellom/microbiomes.

Protein-coding annotation identifiers (COG, EC, KO, and Pfam) were counted for metagenomes with gff3-format annotation files resulting from the IMG annotation pipeline v.5.0.0 or newer, which was 5,683 out of the total 12,063 plotted metagenomes. Gene annotation file formats prior to pipeline v.5.0.0 were not amenable to our analysis methods. These annotation counts were normalized to the total number of assembled bases for each metagenome and used as heatmap “Map Values” to be overlaid on top of metagenome data points. Statistical tests were calculated with the Python package Pingouin (104). In the interactive tool, each heatmap “Map Value” was multiplied by a constant of 10^9^ to yield an appropriate Matplotlib (47) reverse cividis colormap value implemented in Bokeh. Metagenomes without gff3-format annotation files were assigned a heatmap “Map Value” of “NaN” and are hidden from the protein annotation view of the interactive tool. Since protein-coding annotations can have a wide range of counts depending on the gene, the interactive tool “Heat” slider can adjust the heatmap color values median to better illuminate ubiquitously lower or higher counts.

With Scikit-learn’s RandomForestClassifier, we used a random forest model with a 75%/25% training/testing data split on the metagenome GOLD and KNN Ecosystem Type classifications with protein-coding annotations to extract an ordered list of gene annotation importances according to mean decrease in impurity (105) (File S2). Gene annotation importances from KNN Ecosystem Type classifications were derived from our tetranucleotide analysis methods described here, while gene annotation importances from GOLD Ecosystem Type classifications were derived from environmental annotation in GOLD. These ordered lists were used to identify top KNN Ecosystem classification-specific gene annotations.

## SUPPLEMENTAL MATERIAL

File S1 msystems.01744-24-s0001.xlsx Metagenome data table downloaded from the metagenome stability diagram tool.

File S2 msystems.01744-24-s0002.xlsx Ranked gene identifier random forest importances.

File S3 msystems.01744-24-s0003.xlsx Metagenome data table downloaded from the metagenome stability diagram tool.

Figure S1 msystems.01744-24-s0004.pdf Effect of KNN *k* value on Scikit-learn classification report metrics.

Supplemental Text msystems.01744-24-s0005.docx Captions of all supplemental material and link to File S4.

ASM does not own the copyrights to Supplemental Material that may be linked to, or accessed through, an article. The authors have granted ASM a non-exclusive, world-wide license to publish the Supplemental Material files. Please contact the corresponding author directly for reuse.
