# Supplementary material for: Tetranucleotide frequencies differentiate genomic boundaries and metabolic strategies across environmental microbiomes
Source: mSystems. 2025 Jul 8;10(8):e01744-24. doi: 10.1128/msystems.01744-24 (PMC12363243; doi:10.1128/msystems.01744-24)
Supplement: Figure S1 — Effect of KNN k value on Scikit-learn classification report metrics. [file msystems.01744-24-s0004.pdf]

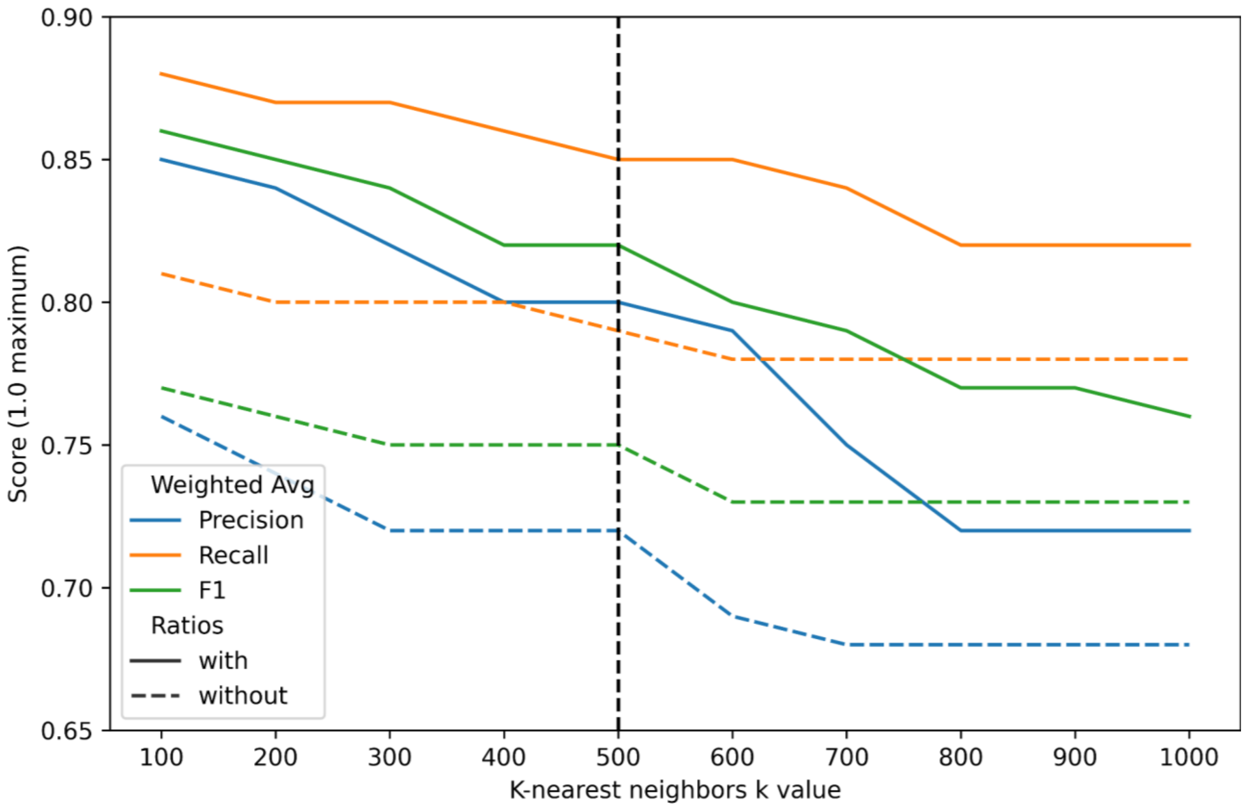

Figure S1: Scikit-learn classification report metrics for k-nearest neighbors k=100 to k=1,000, in increments of 100. Data lines for precision (blue), recall (orange), and F1 (green) scores are plotted with (solid line) and without (dashed line) the inclusion of ratios of tetranucleotide frequencies at a training/test split of 80%/20%. The dashed line at k=500 indicates the k value used in our analysis.
