# Supplementary material for: Tetranucleotide frequencies differentiate genomic boundaries and metabolic strategies across environmental microbiomes
Source: mSystems. 2025 Jul 8;10(8):e01744-24. doi: 10.1128/msystems.01744-24 (PMC12363243; doi:10.1128/msystems.01744-24)
Supplement: Supplemental Text — Captions of all supplemental material and link to File S4. [file msystems.01744-24-s0005.docx]

**Supplemental Material**

**Supplemental File S1 - Supplemental_File_S1.xlsx**

Metagenome data table downloaded from the metagenome stability diagram tool, including IMG Taxon IDs, LDA coordinates, plotting colors, KNN Ecosystem classifications (map_classification), IMG Analysis Project IDs, GOLD Analysis IDs, GOLD Ecosystem classifications. A metagenome count summary for each GOLD Ecosystem and KNN Ecosystem classification is included.

**Supplemental File S2 - Supplemental_File_S2.xlsx**

Ranked gene identifier random forest importances as calculated by mean decrease in impurity for all KNN and GOLD Ecosystem classification as well as overall across all metagenomes.

**Supplemental File S3 - Supplemental_File_S3.xlsx**

Metagenome data table downloaded from the metagenome stability diagram tool, including IMG Taxon IDs, Normalized gene abundance values (map_value), LDA coordinates, plotting colors, KNN Ecosystem classifications (map_classification), IMG Analysis Project IDs, GOLD Analysis IDs, GOLD Ecosystem classifications for “KO:K00368; *nirK*”, “KO:K03385; *nrfA*”, and “KO:K00362; *nirB*” gene abundances in **Figure 5**.

**Supplemental File S4 - Supplemental_File_S4.csv**

Tetranucleotide frequencies and ratios of tetranucleotide frequencies of every metagenome included in the metagenome stability diagram tool. Available at https://doi.org/10.5061/dryad.tb2rbp0c8.

**Supplemental Figure S1 - Supplemental_Figure_S1.pdf**

Supplemental figure depicting the effect of KNN k value on Scikit-learn classification report metrics precision, recall, and F1 scores, with and without the inclusion of ratios of tetranucleotides in the data set.
